# Caffeic acid suppresses cyclin D1 expression by directly binding to ribosomal protein S5 in colorectal cancer cells

**DOI:** 10.1038/s41598-026-42196-6

**Published:** 2026-03-05

**Authors:** Motoki Watanabe, Shogen Boku, Mamiko Sukeno, Kaito Kobayashi, Tomoshi Kameda, Yosuke Iizumi, Wataru Nishio, Michihiro Mutoh, Toshiyuki Sakai

**Affiliations:** 1 Department of Molecular-Targeting Prevention, Kyoto Prefectural University of Medicine, Kyoto, Japan; 2 Department of Clinical Oncology, Kansai Medical University Hospital, Osaka, Japan; 3 Department of Drug Discovery Medicine, Kyoto Prefectural University of Medicine, Kyoto, Japan; 4 Artificial Intelligence Research Center, National Institute of Advanced Industrial Science and Technology (AIST), Tokyo, Japan

**Keywords:** Caffeic acid, RPS5, Cyclin D1, Colorectal cancer, Biochemistry, Cancer, Cell biology, Molecular biology

## Abstract

Colorectal cancer (CRC) is a leading cause of cancer-related mortality worldwide, and dietary components such as coffee have been epidemiologically associated with a reduced risk of CRC. However, the molecular mechanisms underlying this effect remain elusive. In this study, we found that caffeic acid, a hydrolysate of chlorogenic acid abundant in coffee, significantly suppressed colony formation in human CRC cells. Chemical pull-down assays using nano-magnetic beads combined with mass spectrometry identified ribosomal protein S5 (RPS5) as a direct binding target of caffeic acid. Molecular dynamics simulations further supported the stability of the interaction between caffeic acid and a specific binding pocket on RPS5. Mechanistically, RNA interference-mediated knockdown of RPS5 induced G1 cell cycle arrest and downregulated cyclin D1 expression at both mRNA and protein levels, without affecting its promoter activity, suggesting a post-transcriptional regulatory mechanism of cyclin D1 by RPS5. These findings reveal a previously unrecognized RPS5-cyclin D1 axis targeted by caffeic acid and provide novel mechanistic insights into the potential chemopreventive effects of coffee against CRC.

**Supplementary Information:**

The online version contains supplementary material available at 10.1038/s41598-026-42196-6.

## Introduction

The incidence of colorectal cancer (CRC) is progressively increasing globally, and the International Agency for Research on Cancer predicts that the number of new cases of CRC will increase by approximately 63% to 3.2 million per year by 2040^1^, further increasing the significance of the prevention of CRC. As diet and lifestyle affect the risk of developing CRC, attention has focused on food and drink habits as a reasonable preventive intervention. Coffee, among other foods, is one of the most familiar foods whose regular consumption has been associated with a reduced risk of CRC. For example, a meta-analysis including data from 19 prospective cohort studies, involving approximately 2.04 million people, reported a 7% reduction in the risk of colon cancer for every four cups of coffee consumed daily^2^. Another meta-analysis of 25 case-control studies reported that coffee consumption was associated with a reduced risk of CRC^3^. These epidemiological studies suggest that regular coffee consumption has a specific protective effect against CRC.

However, the specific components of coffee and the molecular mechanisms by which they prevent CRC remain unclear and warrant more precise and transparent elucidation. Considering that many epidemiological studies have shown a reduced risk of CRC even with decaffeinated coffee^4–6^, other components, rather than caffeine, are suggested to play a role in suppressing CRC. Among the ingredients in coffee, chlorogenic acid, which has recently attracted attention as a polyphenol with potent antioxidant properties, has been reported to attenuate DNA damage caused by oxidative stress in human CRC cells^7^. Conversely, chlorogenic acid generates reactive oxygen species, leading to cell-cycle arrest in S phase and activation of the tumor suppressor p53 protein^8^. However, because chlorogenic acid is heat-sensitive and is primarily hydrolyzed in the colon, it is uncertain to what extent chlorogenic acid itself contributes to the preventive effect against CRC.

In this study, we found that caffeic acid, a hydrolysate of chlorogenic acid, suppresses the growth of human CRC cells. Using chemical biology, we identified two caffeic acid-binding proteins, ribosomal protein S5 (RPS5) and prohibitin 2 (PHB2). We focused on RPS5 because its expression is strongly associated with poor prognosis in CRC and because the oncogenic roles of ribosomal proteins are increasingly recognized^9–11^. We therefore investigated the mechanism by which caffeic acid suppresses cancer cell growth through targeting RPS5.

## Materials and methods

### Reagents and chemicals

Caffeic acid and quinic acid were obtained from Sigma-Aldrich (St Louis, MO, USA). iCRT14 was obtained from Selleck (Houston, TX, USA). All reagents were dissolved in dimethyl sulfoxide (DMSO) to make stock solutions. Purified recombinant human PHB2 (TP760501) was obtained from OriGene (Rockville, MD, USA). Purified recombinant human RPS5 (ab137146) was obtained from Abcam (Cambridge, UK). Primary antibodies used in the experiments include rabbit anti-PHB2 antibody (#14085; Cell Signaling Technology, MA, USA), rabbit anti-RPS5 antibody (ab58345; Abcam), mouse anti-His-Tag antibody (#2366; Cell Signaling Technology), rabbit anti-MEK1/2 antibody (#9122; Cell Signaling Technology), rabbit anti-p44/42 MAPK (ERK1/2) antibody (#9102; Cell Signaling Technology), rabbit anti-phospho-p44/42 MAPK (ERK1/2) antibody (#9101; Cell Signaling Technology), mouse anti-cyclin D1 antibody (K0062-3; MBL, Nagoya, Japan), and mouse anti-α-tubulin antibody (CP06; Calbiochem, CA, USA).

### Cell lines and culture

The human colorectal cancer (CRC) cell lines HCT-15 and HCT116 were obtained from the NCI Developmental Therapeutics Program as part of the NCI-60 cell line collection. All cell lines were confirmed negative for mycoplasma infection using the MycoAlert Mycoplasma Detection Kit (Lonza Rockland, ME, USA). HCT-15 cells were cultured in RPMI-1640 supplemented with 10% fetal bovine serum, 2 mM glutamine, 50 U/ml of penicillin, and 100 µg/ml of streptomycin. HCT116 cells were cultured in Dulbecco’s modified Eagle’s medium (DMEM) supplemented with 10% fetal bovine serum (FBS), 4 mM glutamine, 50 U/ml penicillin, and 100 µg/ml streptomycin. Cells were incubated at 37 °C in a humidified atmosphere containing 5% CO_2_.

### Colony formation assay

Colony Formation Assay was performed as previously described^12^ as follows. Briefly, cells were seeded at 200 cells per well in 6-well plates. Seeded cells were incubated for 24 h in 6-well plates and treated with the indicated agents. After around 2 weeks of incubation, the cells were fixed with 10% formalin and stained with 0.1% crystal violet. The area of stained colonies was quantified using ImageJ, a software developed by the US NIH (Bethesda, MD, USA, https://imagej.nih.gov/ij/).

### Preparation of caffeic acid-fixed magnetic beads

Immobilization of caffeic acid onto magnetic beads was performed as previously described^13^ as follows. Briefly, FG beads were purchased from Tamagawa Seiki (Nagano, Japan). Caffeic acid was fixed onto beads with epoxy linkers (TAS8848N1110). The beads were incubated at 37 °C for 24 h with 100 mM caffeic acid in dimethylformamide (DMF) containing potassium carbonate. The next day, the beads were washed with Milli-Q water after cleaning with DMF.

### Purification and identification of caffeic acid-binding proteins

Purification and identification of caffeic acid-binding proteins were previously described^13^ as follows. Briefly, HCT-15 cells were lysed in binding buffer [50 mM Tris-HCl, 150 mM NaCl, 1% NP-40, 1 mM DTT, and 0.43 mM ABSF] at 4 °C for 30 min, then centrifuged. The supernatants were used as whole-cell extracts of HCT-15 cells. The extracts were incubated with agent-immobilized or empty beads at 4 °C for 4 h and then washed three times with binding buffer. Bound proteins were eluted with Laemmli dye and subjected to SDS-PAGE and Western blotting. Proteins were stained with aqueous AgNO_3_, and each strip containing a protein was cut out and digested with Sequencing Grade Modified Trypsin (Promega, Madison, WI, USA). After in-gel digestion, the peptide fragments from each strip were analyzed using an Autoflex II mass spectrometer (Bruker Daltonics, Billerica, MA, USA).

### Bioinformatics analysis

Kaplan-Meier curves were generated using the LOGpc (Long-term Outcome and Gene Expression Profiling Database of pan-cancers) from the Biomedical Informatics Institute (http://bioinfo.henu.edu.cn/Index.html). The LOGpc tool was used to analyze the association between PHB2 expression and prognosis, with the upper quartile of PHB2 expression used to dichotomize PHB2 profiles. The GSE17537 dataset provides cancer prognosis and microarray data, including PHB2 expression profiles of CRC samples. Survival curves were compared using a log-rank test.

### Molecular dynamics simulations of RPS5-caffeic acid binding

The structure of RPS5 was derived from the human 80 S ribosome structure (PDB code: 6qzp). The relaxed structure generated by molecular dynamics (MD) simulation was used for molecular docking calculations, as previously described^14^. Molecular docking simulations were performed using the Smina software^15^, and 10 docking poses of RPS5 and caffeic acid were generated. MD simulations of 10 ns were performed 10 times for each docking pose. RPS5 was described using the AMBER ff14SB force field^16^. Caffeic acid was defined using the GAFF force field^17^ with restrained electrostatic potential (RESP) charges^18^. Water molecules were characterized using the TIP3P model^19^. The docking pose of RPS5 and caffeic acid was protonated and placed in a dodecahedral box. The box size was determined to ensure that all molecules were placed at least 1.5 nm from the box edges. The periodic boundary conditions were applied in all directions. The box was filled with water molecules. Sodium and chloride ions were added to each box to neutralize the total charge and to establish an ion density of 100 mM. After energy minimization, constant-pressure, constant-temperature MD simulations were performed to equilibrate the system at 1 bar and 300 K for 200 ps. Position restraints were applied to Cα atoms of the proteins and all heavy atoms of the caffeic acid during equilibration. The production runs were performed for 10 ns at 300 K. The Berendsen method^20^ and the Parrinello-Rahman method^21^ were used to maintain pressure during equilibration and production, respectively. The covalent bonds of hydrogen atoms were constrained using the LINCS method, and the integration time step was 2.0 fs. MD simulations were performed by using GROMACS 2022.4^22^. The figures were generated using PyMOL.

### Molecular dynamics simulations of RPS5-AU-rich elements binding

The structure of RPS5 was obtained from the human 80 S ribosome structure (PDB code: 6QZP). The AU-rich elements (AREs) sequence, UUAUUUAUUUAUUUAUUUA, which contains multiple AUUUA motifs, was derived from the cyclin D1 mRNA. Initial preparation and analysis of the AREs sequence were performed using AmberTools22^23^. The protein and RNA were simulated in an explicit solvent environment. The system consisted of one RPS5 molecule, one AREs RNA fragment, and approximately 18,300 water molecules, enclosed in a dodecahedral simulation box with a side length of 14.51 nm. Sodium ions were added to neutralize the system’s total charge. The initial distance between the RPS5 protein and the RNA molecule was set to 7.0 nm. Coarse-grained molecular dynamics (MD) simulations were performed using the MARTINI 2.1 force field^24–26^ for both the protein and RNA. After energy minimization, the system was equilibrated under constant pressure and temperature (NPT) conditions at 1 bar and 300 K for 50,000 steps. Production simulations were subsequently conducted for 400,000 steps. Pressure coupling during the NPT simulations was achieved using the Parrinello-Rahman barostat^21^ with a coupling time constant of 12 ps. In comparison, temperature was controlled using the velocity-rescale (V-rescale) thermostat with a coupling time constant of 1 ps^27^. The integration time step was set to 15.0 fs. All simulations were performed using GROMACS version 2022.5^22^.

### RNAi

Oligonucleotides for siRNA targeting RPS5 were obtained from Thermo Fisher Scientific (Waltham, MA, USA). The following siRNAs were used: siRPS5 #1 (HSS109357; Stealth siRNAs), 5’-GGAGCACCGAUGAUGUGCAGAUCAA-3’; siRPS5 #2 (HSS184431; Stealth siRNAs), 5’-GCCGCAACAACGGCAAGAAGCUCAU-3’; negative control siRNA (12935112; Stealth RNAi™ siRNA Negative Control Med GC Duplex #2). Only RNA sequences of the sense strands are shown. Cells were transfected with 10 nM siRNA using the Lipofectamine RNAiMAX Reagent (Invitrogen, Carlsbad, CA, USA) as previously described^28^.

### Cell viability assay

Cell Viability Assay was previously described^29^ as follows. Briefly, the number of viable cells was measured using the Cell Counting Kit-8 assay (Dojindo, Kumamoto, Japan). Briefly, cells were seeded at 2,500 cells per well in 96-well plates. After cells were incubated with each agent or siRNA, the WST-8 reagent from the kit was added to the medium, and the sample was incubated for 4 h. Absorbance at 450 nm was measured using a multi-plate reader (Molecular Devices, CA, USA).

### RNA-Seq

RNA-seq was performed as previously described^14^ as follows. Total RNA was isolated from cells in triplicate, treated with each siRNA using Sepasol-RNA I (Nacalai Tesque) according to the manufacturer’s instructions, and processed using a TruSeq Stranded mRNA sample prep kit (Illumina, San Diego, CA, USA). Poly(A) RNA libraries were constructed using the TruSeq Stranded mRNA library preparation kit (Illumina) and sequenced in 100-bp paired-end reads on the Illumina NovaSeq 6000 platform. Sequencing data were deposited into the DNA Data Bank of Japan Sequence Read Archive (accession nos. DRR356490 - DRR356498).

### Transcriptomics analysis

Transcriptomics of the RNA-seq data was analysed as previously described^14^ as follows. RNA-Seq reads were quantified using ikra (v.2.0.1)^30^, an RNA-Seq pipeline centered on Salmon^31^. Ikra automated RNA-Seq data analysis, including read quality control (sra-tools v.2.10.9), read trimming (Trim Galore v.0.6.7^32^ with Cutadapt v.3.2^33^), transcript quantification (Salmon v.1.4.0; reference transcript sets from GENCODE release 37 for humans), and tximport v.1.6.0. These tools were used with their default parameters. Count tables were imported into Integrated Differential Expression and Pathway Analysis (iDEP v.2.01^34^), an integrated web application for Gene Ontology (GO) analysis of RNA-Seq data. Absolute fold changes > 2 were considered enriched and investigated further by Metascape^35^. P values < 0.05 were used to determine which functions could be used for further investigation.

### Protein isolation and western blotting

Protein isolation and Western blotting were performed as previously described^36^ as follows. Briefly, cells were lysed with buffer containing 50 mM Tris-HCl, 1% SDS, 1 mM dithiothreitol (DTT), and 0.43 mM 4-(2-aminoethyl) benzenesulfonyl fluoride hydrochloride (ABSF). The lysates were sonicated and centrifuged at 20,400 *g* for 20 min at 4 °C, and the supernatants were collected. Equal amounts of protein extract were subjected to SDS-PAGE and transferred to a PVDF membrane (EMD Millipore, Billerica, MA, USA). After being probed with primary antibodies and HRP-conjugated secondary antibodies, immunoreactive bands were detected with Chemi-Lumi One L (Nacalai Tesque) or Immobilon Western Chemiluminescent HRP Substrate (EMD Millipore).

### Cell cycle analysis

Cell cycle analysis was performed as previously described^37^ as follows. Briefly, cells were seeded at 50,000 cells per well in 6-well plates and incubated for 24 h. The cells were then treated with siRNA and harvested by trypsinization. After centrifugation, cells were suspended in PBS containing 0.1% Triton X-100 and 25 µg/ml propidium iodide. Stained cells were analyzed using FACSCalibur (Becton Dickinson, NJ, USA). Cell cycles were analyzed using Modfit LT software (Becton Dickinson).

### RNA isolation and quantitative PCR

RNA isolation and quantitative PCR were performed as previously described^38^ as follows. Briefly, total RNA was isolated from cells treated with siRNAs using Sepasol-RNA I (Nacalai Tesque) according to the manufacturer’s instructions. Total RNA (2 µg) was reverse-transcribed to complementary DNA (cDNA) in a 20 µL reaction volume with MMTV-reverse transcriptase (Promega) and oligo (dT) primers (Toyobo, Osaka, Japan). An equivalent volume of cDNA solution was used for quantitative PCR. cDNA was amplified using an ABI 7300 real-time PCR system (Applied Biosystems, Foster City, CA, USA) with TaqMan Probes for *CCND1* (Hs00765553_m1) and *GAPDH* (Hs02758991_g1) (Applied Biosystems). The expression of cyclin D1 mRNA was normalized to that of GAPDH mRNA in the same sample.

### Plasmid DNA transfection and luciferase assay

Plasmid DNA transfection and luciferase assay were performed as previously described^13^ as follows. The full-length promoter plasmid of cyclin D1 (-962CD1) and an empty plasmid (pGL3 Basic) were gifts from O. Tetsu, UCSF Helen Diller Family Comprehensive Cancer Center at San Francisco. 0.1 µg of each plasmid and 10 nM of each siRNA were co-transfected into HCT-15 cells using Lipofectamine 2000 (Invitrogen). After 48 h, cells were lysed with MelioraStar-LT (TOYO INK, Tokyo, Japan), and luciferase activity in the cell lysate was measured using a luminometer (Berthold Technologies). The obtained data were normalized to the absorbance measured by a Cell Counting Kit-8 assay performed before cell lysis.

### Statistical analysis

All data are presented as the mean ± standard deviation (SD). The significance of differences in the means was tested using a two-tailed unpaired Student’s *t*-test. A P-value < 0.05 was considered statistically significant relative to each control.

## Results

### Caffeic acid, rather than quinic acid, inhibits the colony formation of colorectal cancer cells

**Fig. 1:**
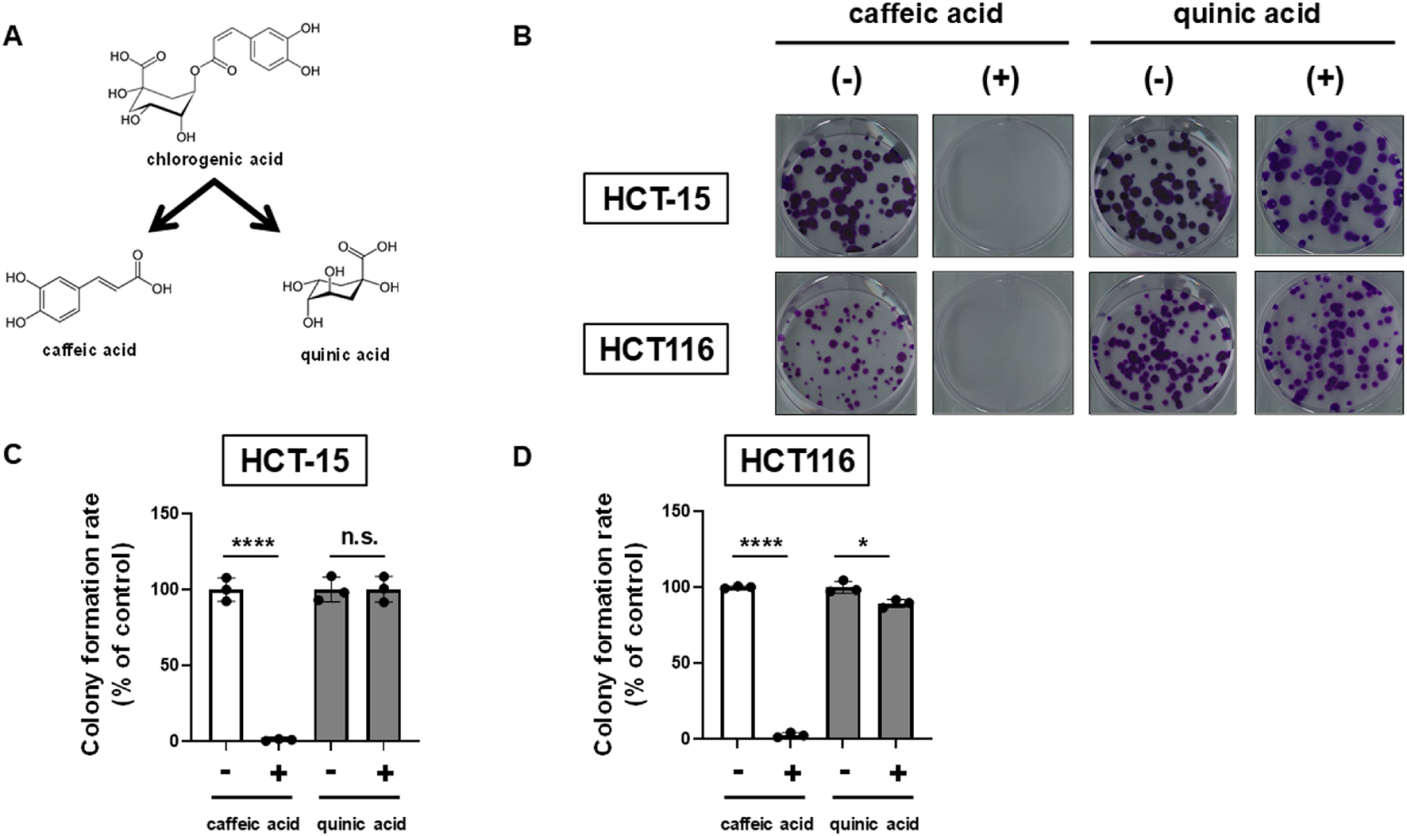
Caffeic acid, rather than quinic acid, inhibits the colony formation of colorectal cancer (CRC) cells. (**A**) Chlorogenic acid and its hydrolysis products, caffeic acid and quinic acid. (**B**) Effects of caffeic acid and quinic acid on the colony formation of CRC cells. HCT-15 and HCT116 cells were treated with 500 µM caffeic acid and quinic acid. After further incubation, colonies were fixed and stained with crystal violet. The representative images of stained colonies are shown. (**C**-**D**) Colony formation rate of cells treated with caffeic acid and quinic acid. HCT-15 cells (**C**) and HCT116 cells (**D**) were treated with each compound at 500 µM. Columns, means (*n* = 3); bars, SD; ns, *P* > 0.1; *, *P* < 0.05; ****, *P* < 0.0001, significantly different from the DMSO-treated control, using a two-tailed unpaired Student’s *t*-test. The experiments were independently repeated twice.

Chlorogenic acid, which has been focused on as a bioactive component in coffee, is rapidly hydrolyzed into caffeic acid and quinic acid (Fig. 1A). We therefore hypothesized that either caffeic acid or quinic acid may show anti-tumor activity. We then treated human colorectal cancer (CRC) cell lines HCT-15 and HCT116 with caffeic acid and quinic acid. While quinic acid had almost no effect on the colony formation of either cell line, caffeic acid nearly eliminated both colonies (Fig. 1B-D), suggesting that caffeic acid, among the ingredients contained in coffee, plays an essential role in the anti-tumor effect against CRC cells.

### Caffeic acid directly binds to prohibitin 2 (PHB2) and ribosomal protein S5 (RPS5)

**Fig. 2:**
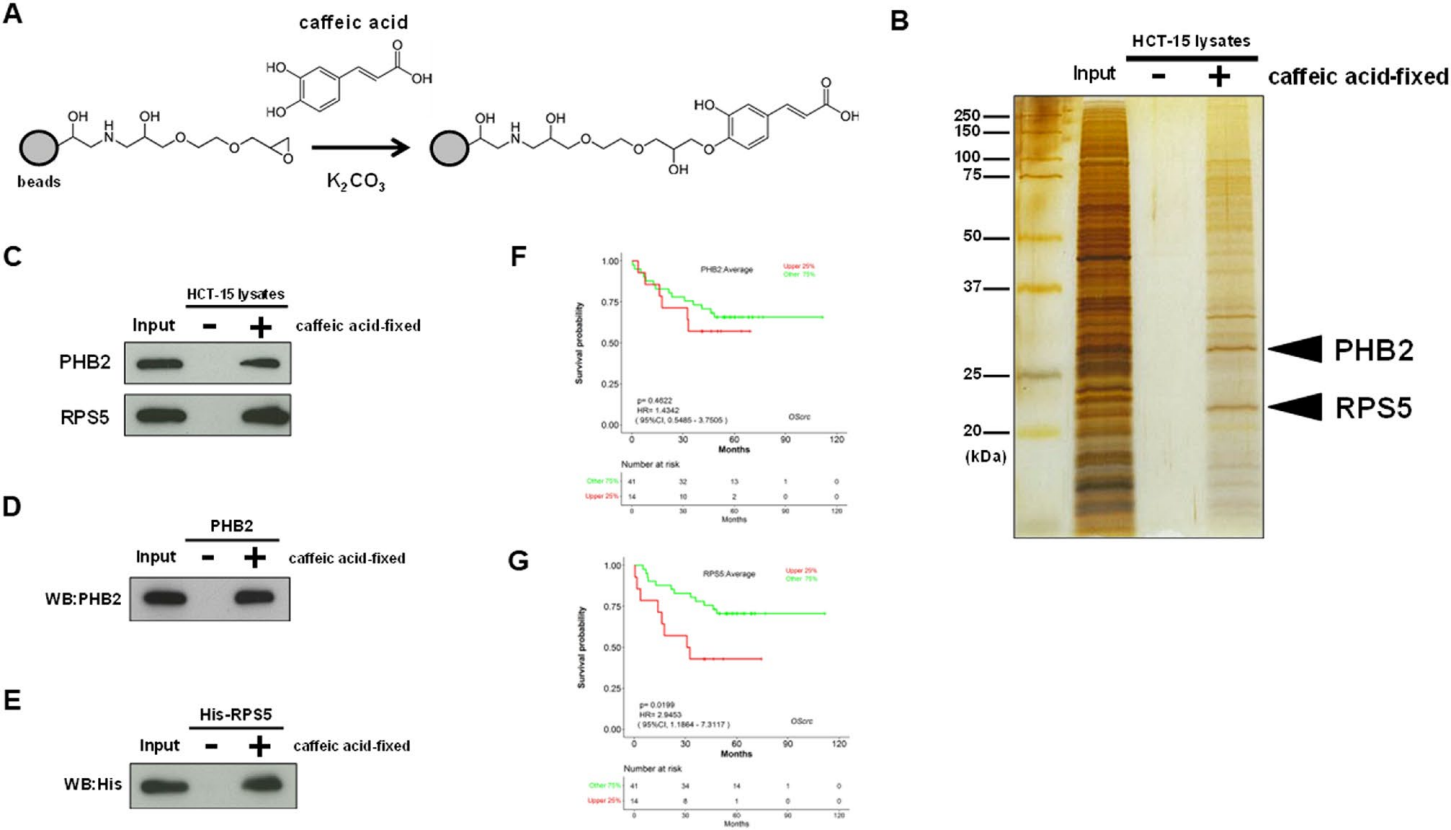
Caffeic acid directly binds to prohibitin 2 (PHB2) and ribosomal protein S5 (RPS5). (**A**) Scheme of immobilization of caffeic acid onto magnetic FG beads and the predicted structure of caffeic acid-fixed beads. (**B**) Identification of caffeic acid-binding proteins. Caffeic acid-binding proteins were purified from whole cell lysates of HCT-15 cells with caffeic acid-fixed FG beads and detected by silver staining. Mass spectrometry analysis identified PHB2 and RPS5 as proteins that bind to caffeic acid. The data shown are representative of two independent experiments. Original uncropped silver-stained gels are presented in Supplementary Fig. 3. (**C**) Validation of the interaction of caffeic acid with PHB2 and RPS5. Bound PHB2 and RPS5 were detected by Western blotting with the specific antibodies for each protein. The data shown are representative of two independent experiments. Original blots are presented in Supplementary Fig. 4.　(**D**-**E**) Validation of the interaction of caffeic acid with recombinant PHB2 and RPS5 tagged with His (His-RPS5). Purified recombinant PHB2 (**D**) and His-RPS5 (**E**) were incubated with caffeic acid-fixed FG beads, and bound PHB2 and His-RPS5 were detected by Western blotting. The data shown are representative of two independent experiments. Original blots are presented in Supplementary Figs. 5 and 6, respectively. (**F**-**G**) Prognostic relevance of PHB2 and RPS5 expression in patients with colorectal cancer (CRC). The correlation between high expression of PHB2 (F) or RPS5 (G) (Upper 25%) and overall survival rates was analyzed in patients with CRC using data (GSE17537) from LOGpc. Kaplan-Meier curves were generated using LOGpc. Survival curves were compared by log-rank test. Figure 2G was reproduced from our previous publication^11^.

Next, to clarify the mechanism of the anti-tumor effect of caffeic acid on CRC cells, we identified the binding proteins of caffeic acid in cells. As previously reported^13^, the phenolic OH group of caffeic acid was covalently bonded to the epoxy ring of the nano-magnetic beads, thus immobilizing the caffeic acid on the beads (Fig. 2A). Subsequently, after a mixture of the caffeic acid-immobilized beads and the whole lysates of HCT-15 cells, the caffeic acid-binding proteins were pulled down and analyzed using MALDI-TOF-MS. We then identified that caffeic acid binds to two distinct proteins, prohibitin 2 (PHB2) and ribosomal protein S5 (RPS5) (Fig. 2B). We then successfully confirmed that caffeic acid binds to PHB2 and RPS5 in the cell lysates of HCT15 by western blotting using each specific antibody (Fig. 2C). Although previous studies reported that caffeic acid binds to MEK^39^ and ERK1/2^40^ by cell-free binding assays, our binding assay using HCT-15 lysates showed the binding between caffeic acid and neither MEK nor ERK1/2 (Supplementary Fig. 1). Furthermore, the binding of caffeic acid to recombinant PHB2 (Fig. 2D) and RPS5 tagged with His (Fig. 2E) proteins was confirmed, indicating that caffeic acid directly binds to these proteins. While high expression of PHB2 did not affect CRC survival probability (hazard ratio [HR], 1.4342; 95% CI, 0.5485 to 3.7505; *P* = 0.4622) (Fig. 2F), we previously reported that RPS5 expression is associated with poor prognosis in CRC^14^(Fig. 2G). Taken together, we subsequently focused on RPS5 for further experiments, as its interaction with caffeic acid appeared to play a more critical role in suppressing CRC cell growth.

#### Prediction of the complex structure of RPS5 and caffeic acid

**Fig. 3:**
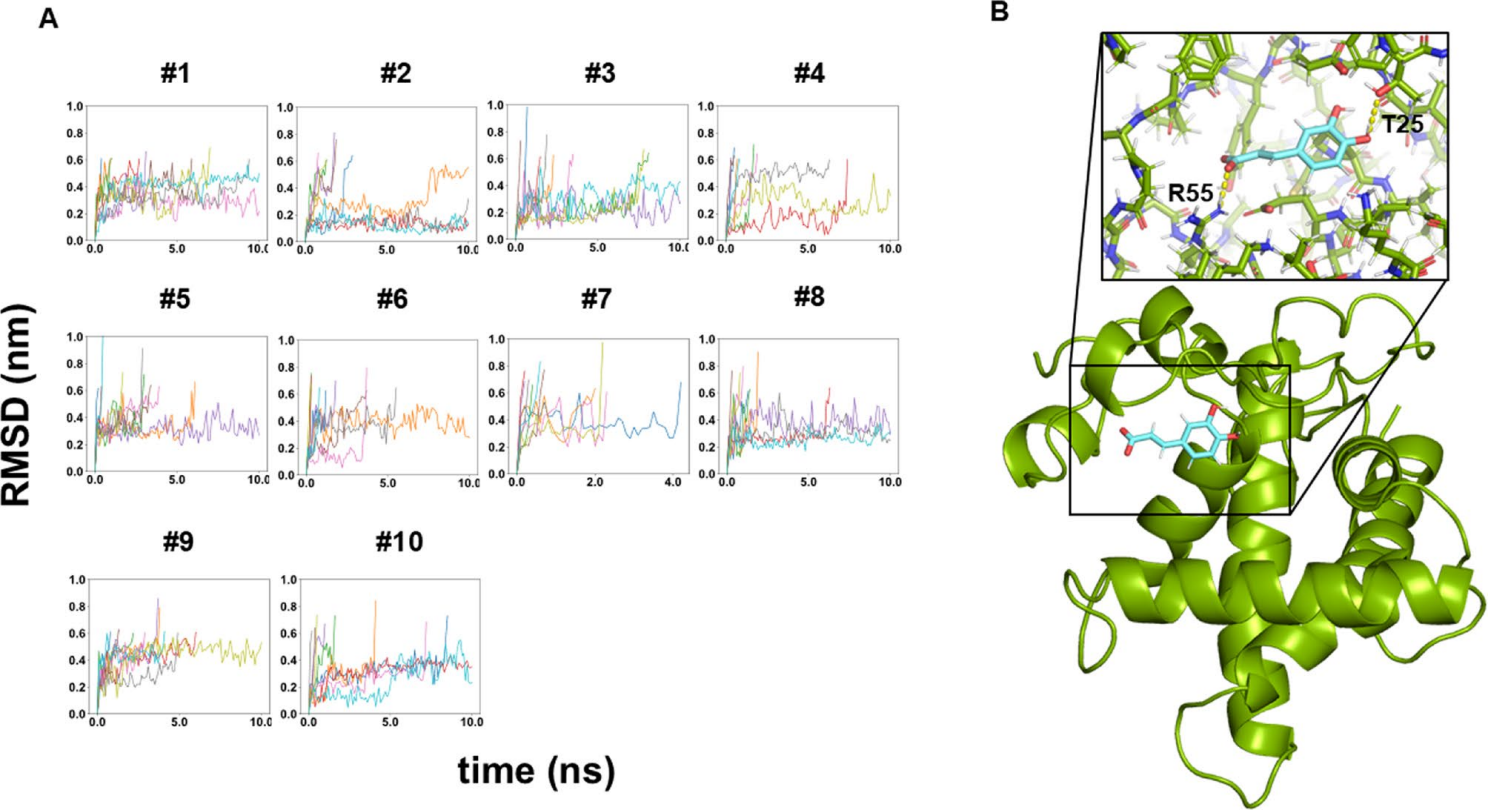
Molecular docking simulation of RPS5 and caffeic acid. (**A**) The root-mean-square deviation (RMSD) values in Molecular dynamics (MD) simulations. The MD simulations of 10 ns were performed 10 times for each of 10 docking poses. (**B**) The candidate complex structure of RPS5 and caffeic acid (pose #2). RPS5 is depicted as a green stick in a cartoon. Caffeic acid is shown as cyan sticks. The yellow dashed lines represent the interactions of RPS5 residues T25 and R55 with caffeic acid.

We then performed the molecular docking simulation to predict the complex structure of RPS5 and caffeic acid. The docking simulation was performed to generate docking poses, and subsequently, a molecular dynamics (MD) simulation was conducted to investigate the stability of these poses. The structures in the trajectories were fitted with the Cα atoms of the protein, and the root-mean-square deviation (RMSD) values of the heavy atoms of the caffeic acid were calculated. When the RMSD value exceeded 0.6 nm, the caffeic acid was considered to have been released from the protein. Among the 10 docking poses, caffeic acid was retained in the highest number of trajectories (4 out of 10) in docking pose #2 (Fig. 3A). The docking structure identified as the most stable by MD simulation (pose #2) revealed that T25 and R55 of RPS5 form a hydrogen bond and a salt bridge, respectively, with caffeic acid (Fig. 3B), both of which are likely to contribute to the stability of this interaction.

### The depletion of RPS5 expression induces G1 cell cycle arrest

**Fig. 4:**
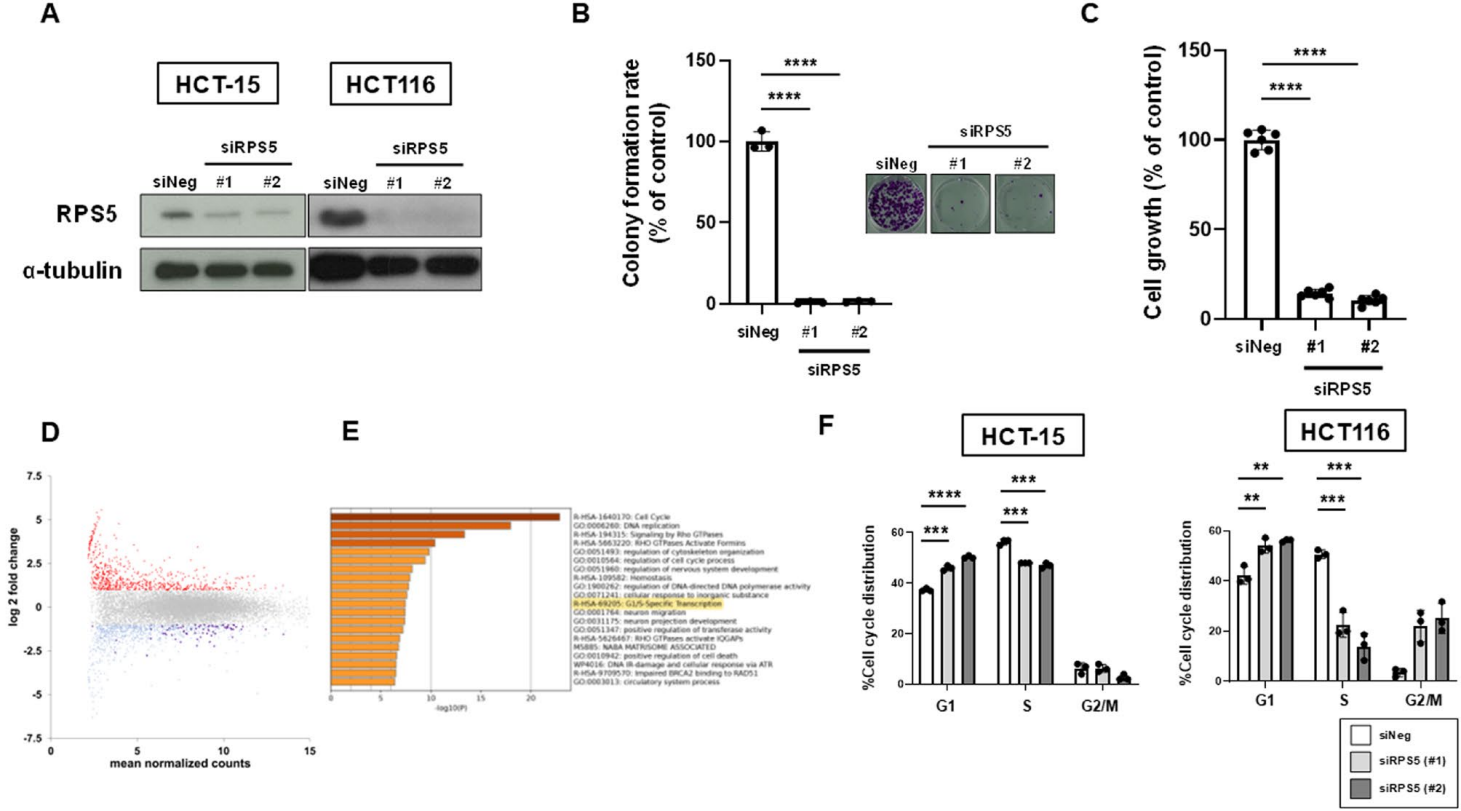
The depletion of RPS5 expression induces G1 cell cycle arrest. (**A**) Knockdown efficacy of siRPS5. Depletion of RPS5 was validated by Western blotting after 48 h siRNA transfection in HCT-15 and HCT116 cells. α-Tubulin was used as a loading control. Original blots are presented in Supplementary Fig. 7. (**B**) Colony formation rate of cells treated with caffeic acid. HCT-15 cells were treated with negative control siRNA (siNeg) or siRPS5. After further incubation, colonies were fixed and stained with crystal violet. The representative images of stained colonies are shown (inset). Columns, means (*n* = 3); bars, SD; ****, *P* < 0.0001, significantly different from the siNeg-transfected sample, using a two-tailed unpaired Student’s *t*-test. The experiments were independently repeated twice. (**C**) Cell viability of RPS5-depleted cells. HCT116 cells were treated with siNeg or siRPS5. Cell viability of siRNA-transfected cells was measured by Cell Counting Kit-8 assay after 72 h transfection. Columns, means (*n* = 6); bars, SD; ****, *P* < 0.0001, significantly different from the siNeg-transfected sample, using a two-tailed unpaired Student’s *t*-test. The experiments were independently repeated twice. (**D**) Differential gene expression analysis between HCT116 cells after 48 h siRPS5 transfection and those of siNeg. In the MA plot, upregulated and downregulated genes in RPS5-depleted HCT116 cells are indicated in blue and red, respectively. The gene set in REACTOME.G1S SPECIFIC TRANSCRIPTION is colored in purple. RNA-seq analysis was performed in HCT116 cells using three independent biological replicates (*n* = 3). (**E**) The top 20 pathways of enrichment analysis with downregulated genes in RPS5-depleted HCT116 cells. (**F**) Cell cycle analysis following siRPS5 treatment. HCT-15 and HCT116 cells were treated with siNeg or siRPS5 for 48 h. The DNA content of the cells was determined by flow cytometry. The distribution of cells in the G1, S, and G2/M phases of the cell cycle is shown. Columns, means (*n* = 3); bars, SD; **, *P* < 0.01, ***, *P* < 0.001, ****, *P* < 0.0001, significantly different from each siNeg-transfected sample, using a two-tailed unpaired Student’s *t*-test. The experiments were independently repeated twice.

We investigated the role of RPS5 in CRC cell growth using siRNAs targeting different sequences of the RPS5 gene. The depletion of RPS5 using siRNA was confirmed in HCT-15 and HCT116 cells (Fig. 4A). The depletion of RPS5 nearly eliminated colony formation (Fig. 4B) of HCT-15 cells, analogous to caffeic acid treatment (Fig. 1C). Furthermore, during short-term incubation, RPS5 depletion resulted in an approximately 85% reduction in cell growth of HCT116 cells (Fig. 4C). As previously reported, RNA-seq data showed differential gene expression following siRPS5 treatment in HCT116 cells^14^. We reanalyzed these data and found that genes involved in the G1-S phase transition were reduced upon RPS5 depletion (Fig. 4D, E). Indeed, RPS5 depletion resulted in an approximately 10%-point increase in cells in the G1 phase compared with the DMSO control, with a significant decrease in cells in the S phase in both HCT-15 and HCT116 cells (Fig. 4F).

#### RPS5 may regulate cyclin D1 at the post-transcriptional level

**Fig. 5:**
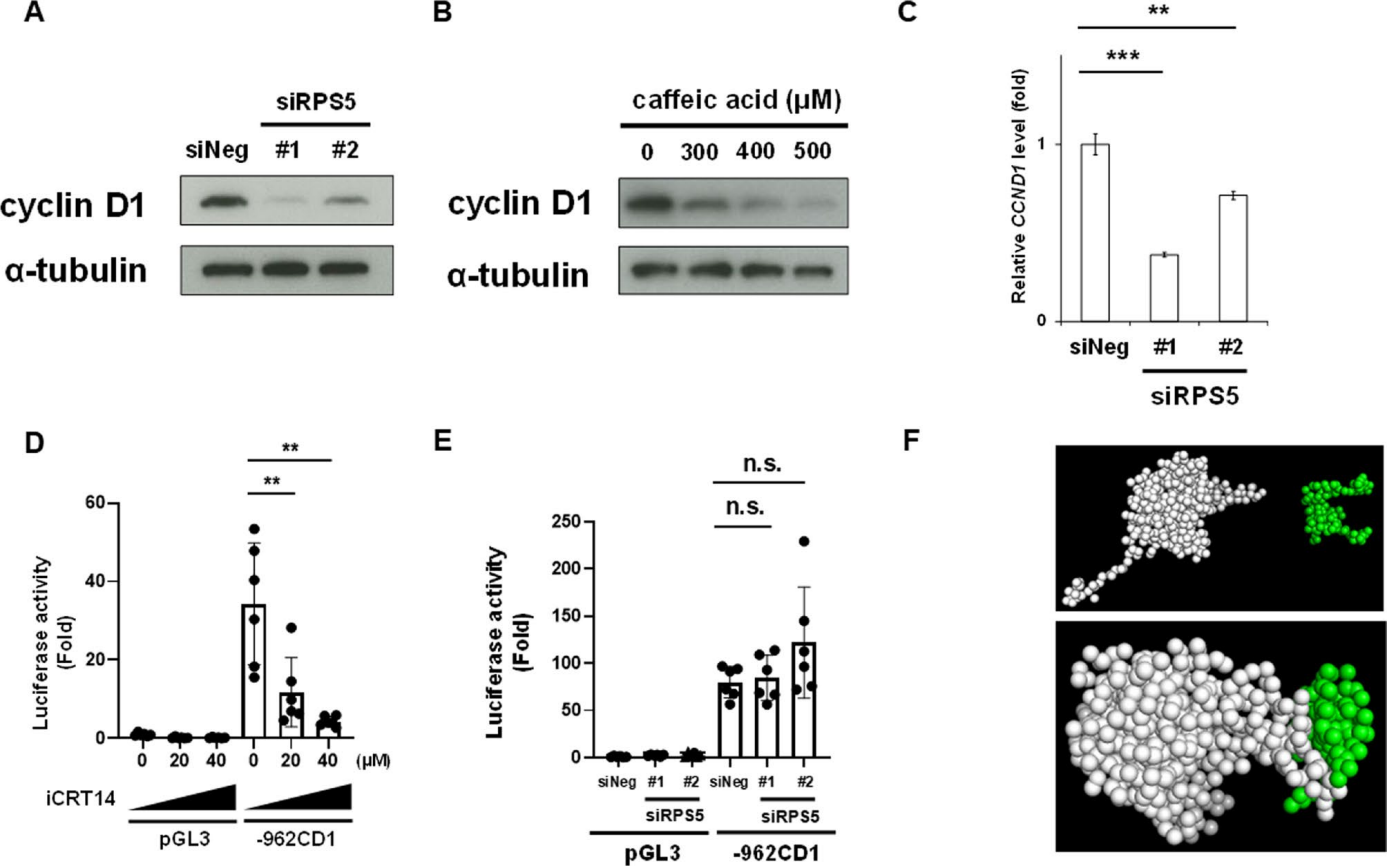
RPS5 may regulate cyclin D1 at the post-transcriptional level. (**A**) Expression of cyclin D1 in RPS5-depleted cells. HCT-15 cells were treated with siNeg or siRPS5 for 72 h. The expression of cyclin D1 was analyzed by Western blotting. α-Tubulin was used as a loading control. The experiments were independently repeated twice. Original blots are presented in Supplementary Fig. 8. (**B**) Expression of cyclin D1 in caffeic acid-treated cells. HCT-15 cells were treated with caffeic acid at the indicated concentrations for 6 days. The expression of cyclin D1 was analyzed by Western blotting. α-Tubulin was used as a loading control. The experiments were independently repeated three times. Original blots are presented in Supplementary Fig. 9. (**C**) Cyclin D1 mRNA (*CCND1*) expression in RPS5-depleted cells. *CCND1* expression was analyzed by quantitative PCR in HCT-15 cells treated with siNeg or siRPS5 for 72 h. *CCND1* mRNA levels were normalized to *GAPDH* mRNA, and data from siNeg treatment were set to 1. Columns, means (*n* = 3); bars, SD; ***, *P* < 0.001, **, *P* < 0.01 significantly different from the siNeg-treated control, using a two-tailed unpaired Student’s *t*-test. The experiments were independently repeated three times. (**D**) Cyclin D1 promoter activity in iCRT14-treated cells. HCT-15 cells were transfected with an empty plasmid (pGL3 Basic) or the promoter plasmid of cyclin D1 (-962CD1). After 24 h of transfection, cells were treated with iCRT14 at the indicated concentrations for 48 h, and luciferase activity was measured. The obtained data were normalized to the absorbance previously measured by a Cell Counting Kit-8 assay. The data obtained from pGL3 Basic and DMSO treatments are set to 1. Columns, means (*n* = 6); bars, SD; **, *P* < 0.01, significantly different from − 962CD1 and DMSO-treated sample, using a two-tailed unpaired Student’s *t*-test. The experiments were independently repeated twice. (**E**) Cyclin D1 promoter activity in RPS5-depleted cells. HCT-15 cells were cotransfected with siNeg or siRPS5 and pGL3 Basic or t-962CD1. After 72 h of transfection, luciferase activity was measured. The obtained data were normalized to the absorbance previously measured by a Cell Counting Kit-8 assay. The data obtained under siNeg and pGL3 Basic treatments are set to 1. Columns, means (*n* = 6); bars, SD; ns, *P* > 0.1, using a two-tailed unpaired Student’s *t*-test. The experiments were independently repeated twice. (**F**) Coarse-grained molecular dynamics (MD) simulations illustrating the association between RPS5 and AU-rich elements (AREs) in RNA. Representative snapshots at the initial state (0 steps, upper panel) and after 400,000 simulation steps (lower panel) are shown. RPS5 is depicted in white, and the AREs RNA fragment in green. Four independent simulations were performed under identical conditions, each initialized with a different set of random initial velocities.

We further analyzed in detail the mechanism by which RPS5 depletion induces G1 arrest. As cyclin D1 is known to play an essential role in cell cycle progression^41^–^43^, we indeed observed a decrease in cyclin D1 following siRPS5 treatment (Fig. 5A). Consistently, the RPS5 ligand caffeic acid also decreased cyclin D1 levels in a dose-dependent manner (Fig. 5B). Considering previous studies reporting caffeic acid binding to MEK^39^ and ERK1/2^40^, as well as the established role of the MAPK pathway in positively regulating cyclin D1 expression^44^,^45^, we assessed the effect of caffeic acid on ERK1/2 phosphorylation and found that caffeic acid did not affect ERK1/2 phosphorylation levels (Supplementary Fig. 2). We subsequently found that RPS5 depletion reduced cyclin D1 mRNA levels, corresponding to an approximately 30–60% reduction compared with siNeg-treated cells (Fig. 5C). We then investigated whether RPS5 depletion suppresses cyclin D1 promoter activity using a full-length cyclin D1 reporter plasmid containing the region from the transcription start site to 962 bp. While iCRT14, an inhibitor of β-catenin binding to the TCF/LEF site, used as a positive control, suppressed cyclin D1 promoter activity in a dose-dependent manner (Fig. 5D), the depletion of RPS5 did not suppress its promoter activity (Fig. 5E), suggesting that RPS5 regulates cyclin D1 through post-transcriptional mechanisms, such as the regulation of mRNA stability. We then hypothesized that RPS5 may directly associate with AU-rich elements (AREs), which are critical for the stability of cyclin D1 mRNA^46^. To test this hypothesis, we performed coarse-grained MD simulations and predicted that RPS5 preferentially interacts with the AREs (Fig. 5F), implying that RPS5 may regulate cyclin D1 expression by modulating mRNA stability at the post-transcriptional level.

## Discussion

In the present study, we identified RPS5 as a novel binding protein of the bioactive component of coffee, caffeic acid, using chemical biology approaches. This discovery revealed a novel mechanism by which caffeic acid suppresses cyclin D1 expression by directly targeting RPS5 in CRC cells. Our study could provide evidence for epidemiological studies indicating a preventive effect of regular coffee consumption on CRC.

In recent years, there has been a growing appreciation of the anti-tumor effects of caffeic acid. Caffeic acid is reported to regulate approximately all of the hallmarks of cancer pleiotropically; suppression of growth signals, angiogenesis, tissue invasion and metastasis, tumor-promoting inflammation, immune evasion, and cancer-specific metabolism^47^–^49^. To essentially understand why a simple structural polyphenol such as caffeic acid could exhibit such diverse anti-tumor effects, it is crucial to understand to what molecules and how caffeic acid binds in cancer cells (or surrounding stromal cells); however, very few studies have identified the direct target molecules of caffeic acid, including from a structural biological perspective. Nam Joo Kang et al. reported that caffeic acid binds to MEK1 and TOPK in an ATP non-competitive manner^39^. On the other hand, Ge Yang et al. showed that caffeic acid binds to the ATP pocket of ERK1/2^40^. These previous reports suggest that caffeic acid inhibits the MAPK cascade in experimental settings using recombinant proteins. However, our pull-down assay using CRC cell lysates showed no detectable binding of caffeic acid to MEK or ERK1/2 (Supplementary Fig. 1). Consistently, caffeic acid did not affect ERK1/2 phosphorylation in cells (Supplementary Fig. 2). These differences likely reflect context-dependent interactions. Unlike these previous studies, we unbiasedly identified RPS5 as a novel target of caffeic acid in CRC cells, supporting its functional relevance in this context.

Much attention is currently being focused on the extra-ribosomal functions of various ribosomal proteins, particularly in relation to cancer^9^–^10^. We previously reported that RPS5 is an oncogenic protein; high RPS5 expression is associated with poor prognosis in CRC and lung cancer^14^. Furthermore, RPS5 suppresses p53 expression in *KRAS*-mutant cancer cells and is involved in resistance to cell death induced by the MEK inhibitor trametinib^14^. RPS5 also binds to and stabilizes cyclin-dependent kinase 6^50^, which is crucial for cell cycle progression. Of particular interest, in these previous studies, we initially identified RPS5 as a target molecule for the natural food components, perillyl alcohol^14^, sesaminol^14^, and fucoxanthinol^50^. The recurrent identification of RPS5 as a binding target of multiple structurally distinct dietary natural compounds, including polyphenols, suggests that RPS5 may function as an “antitumor phytochemical-sensitive regulatory hub” in cancer cells. We are currently investigating, from a structural biology approach, why these structurally uncorrelated phytochemicals, including caffeic acid, commonly bind to RPS5.

Cyclin D1 is a critical molecule in G1-S cell cycle progression^41^–^43^ and is highly expressed in various cancers^51^,^52^. Notably, cyclin D1 is also overexpressed in colorectal polyp lesions^53^. Thus, cyclin D1 plays a crucial role in the carcinogenesis of CRC, and the inhibitory effect of caffeic acid on cyclin D1 expression (Fig. 5B) may be an important mechanism underlying the preventive effect of caffeic acid on CRC. We also demonstrated that the caffeic acid-binding protein RPS5 positively regulates cyclin D1 expression. Depletion of RPS5 suppressed cyclin D1 at mRNA level (Fig. 5C) without affecting cyclin D1 promoter activity (Fig. 5E). Although biochemical assays such as RNA immunoprecipitation or mRNA stability analysis were not performed in the present study, our coarse-grained MD simulations suggest that RPS5 may directly interact with AREs involved in *CCND1* mRNA stability^46^ (Fig. 5F), providing a plausible mechanistic basis for the post-transcriptional regulation of cyclin D1, although the precise mechanism underlying this process remains to be elucidated in future studies.

Our study has several limitations. A significant issue is that relatively high concentrations of caffeic acid (300–500 µM) were required to show antiproliferative effects under in vitro conditions. This requirement may reflect multiple experimental factors, including limited cellular uptake, active efflux, metabolic instability of caffeic acid in culture, and the absence of the complex pharmacokinetic environment present in vivo. Generally, caffeic acid peaks in serum within 1 h after coffee consumption^54^; however, the concentration of free caffeic acid after coffee consumption is unstable and unpredictable, as several factors may modulate its availability in vivo. For example, because caffeic acid is more soluble in water when coexisting with arginine^55^, simultaneous consumption of coffee with arginine-rich food may help maintain caffeic acid concentration under physiological conditions. It is also possible that interaction with RPS5 may contribute to the stabilization of caffeic acid under physiological conditions. Moreover, because dietary caffeic acid is directly exposed to the intestinal lumen, colorectal tissues may experience higher local concentrations than those reflected by plasma measurements. Alternatively, microbiota-dependent processes, including the deconjugation of caffeic acid metabolites in the gastrointestinal tract, may influence the bioavailability of caffeic acid. Taken together, our findings should be interpreted as providing a mechanistic proof of concept that caffeic acid can modulate the RPS5-cyclin D1 axis under defined experimental conditions, rather than as a direct simulation of physiological exposure levels. Future studies will be required to determine whether this mechanism operates at physiologically achievable concentrations, including investigations using more bioavailable metabolites or derivatives, as well as animal models and human clinical studies. Another limitation of this study is that the direct interaction between caffeic acid and RPS5 was experimentally demonstrated using cell lysates (Fig. 2C) and purified recombinant proteins (Fig. 2E), but not directly confirmed in intact living cells. Although MD simulations support a stable and specific interaction between caffeic acid and RPS5 (Fig. 3), these approaches do not fully recapitulate the intracellular environment, where factors such as subcellular localization, molecular crowding, and post-translational modifications may influence binding.

In conclusion, we demonstrated the significance of caffeic acid as a bioactive component responsible for coffee’s inhibitory effect on CRC. Furthermore, using chemical biology and MD simulations, we showed that caffeic acid inhibits RPS5 function by directly binding to it, thereby inhibiting CRC cell growth. We also revealed the mechanism by which RPS5 regulates cyclin D1 and cell cycle progression. Although future studies, including analyses of animal models and human CRC clinical samples, are required, these findings could pave the way for translational approaches such as the structural optimization or derivatization of caffeic acid to improve stability, cellular permeability, and target engagement, thereby enhancing its therapeutic or preventive potential. In parallel, direct inhibition or functional modulation of RPS5 by its ligands may represent a novel strategy for CRC through targeting the RPS5-cyclin D1 axis.

## Supplementary Information

Below is the link to the electronic supplementary material.

Supplementary Material 1

## Data Availability

The RNA sequencing data generated during the current study have been deposited in the DNA Data Bank of Japan Sequence Read Archive (DDBJ SRA) under accession numbers DRR356490-DRR356498. These data are publicly available at https://ddbj.nig.ac.jp/resource/sra-run/DRR356490.
