# Supplementary material for: Caffeic acid suppresses cyclin D1 expression by directly binding to ribosomal protein S5 in colorectal cancer cells
Source: Sci Rep. 2026 Mar 5;16:12965. doi: 10.1038/s41598-026-42196-6 (PMC13096365; doi:10.1038/s41598-026-42196-6)
Supplement: Supplementary file 1 — Supplementary Material 1 [file 41598_2026_42196_MOESM1_ESM.pdf]

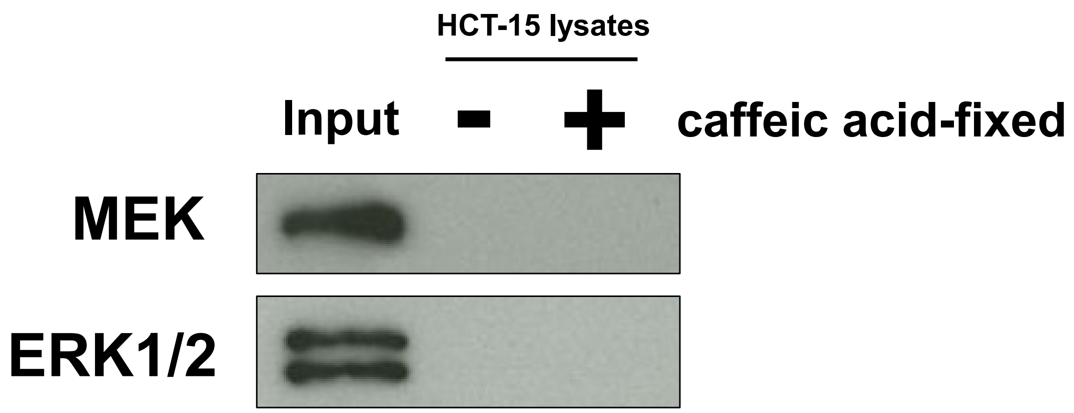

Interaction analysis of caffeic acid with MEK and ERK1/2. No binding of MEK or ERK1/2 to caffeic acid was detected by Western blotting using specific antibodies against each protein. Original blots are presented in Supplementary Fig. 10.

Supplementary Fig. 1

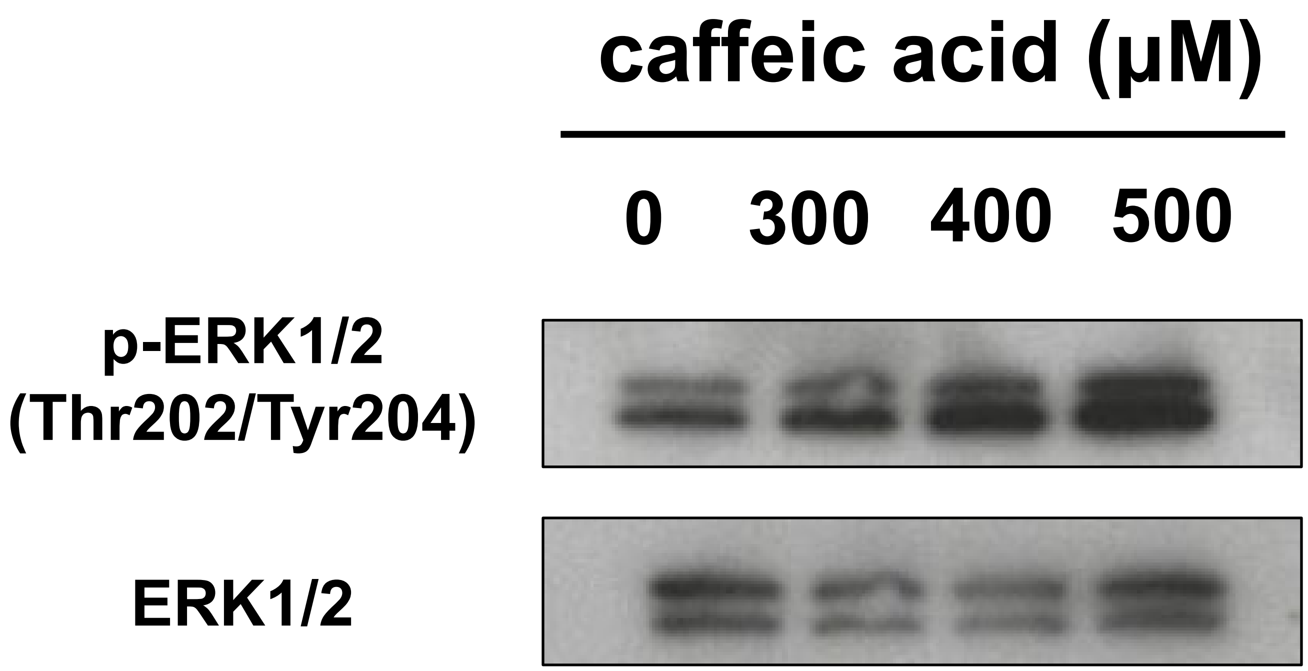

Phosphorylation status of ERK in caffeic acid-treated cells. HCT-15 cells were treated with caffeic acid at the indicated concentrations for 6 days. ERK phosphorylation and total ERK expression were analyzed by Western blotting. Original blots are presented in Supplementary Fig. 11.

Supplementary Fig. 2

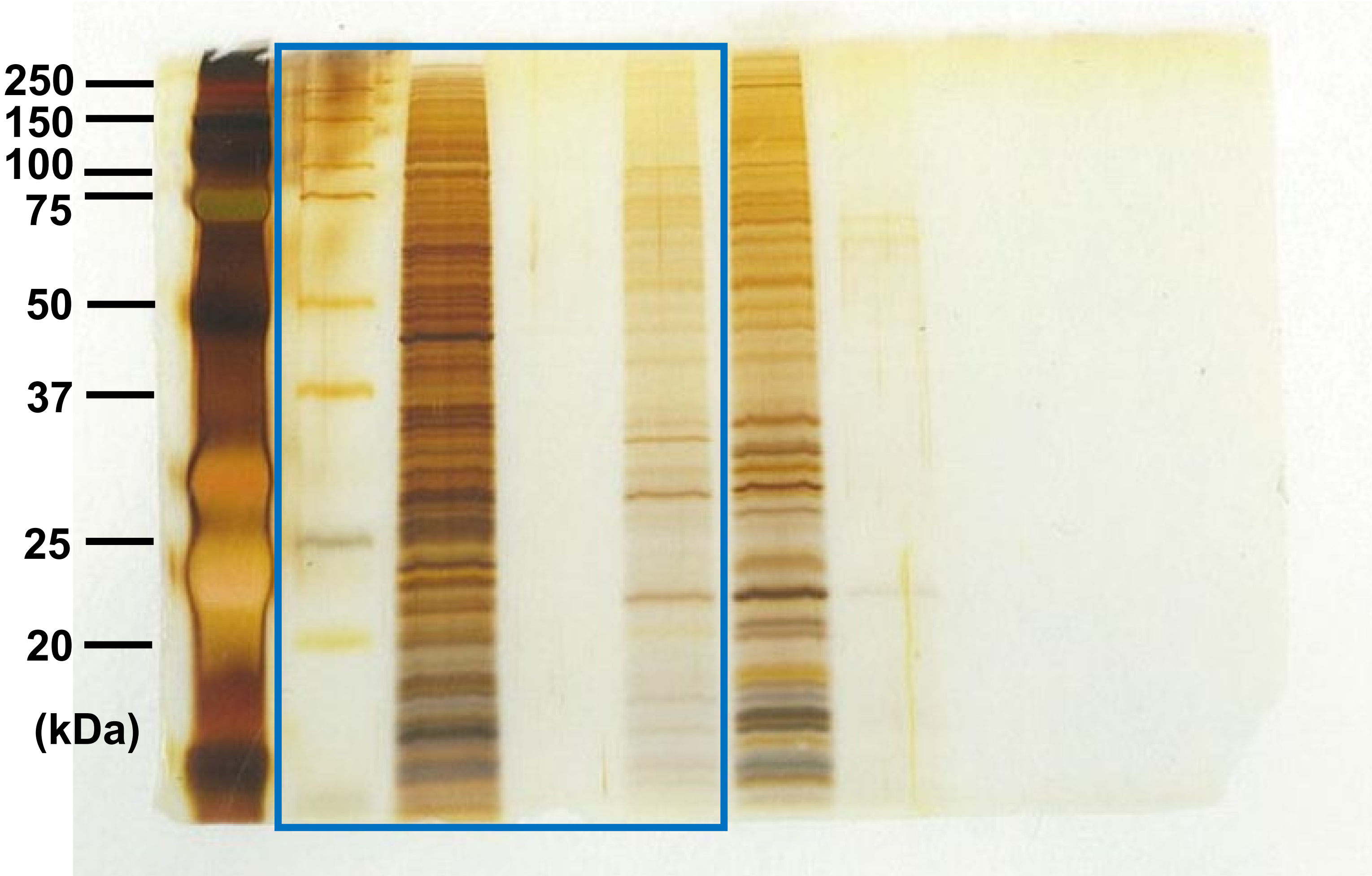

Uncropped silver-stained gel for Figure 2B

Supplementary Fig. 3

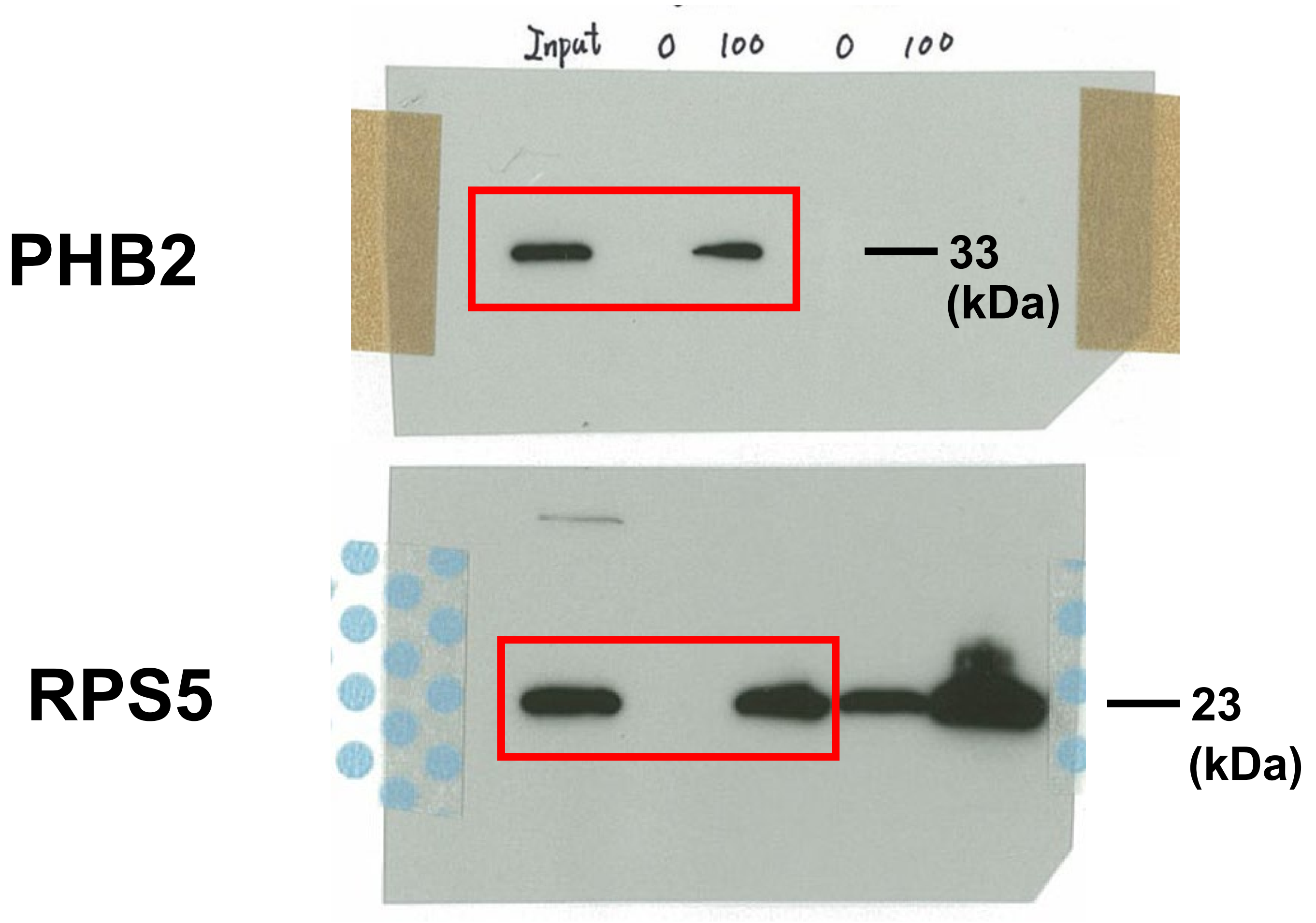

Uncropped western blots for Figure 2C

Molecular weights were estimated using MagicMark™ XP Western Protein Standard (Thermo Fisher Scientific, Waltham, MA, USA) and DynaMarker® Protein MultiColor Stable II (BioDynamics Laboratory Inc., Tokyo, Japan).

Supplementary Fig. 4

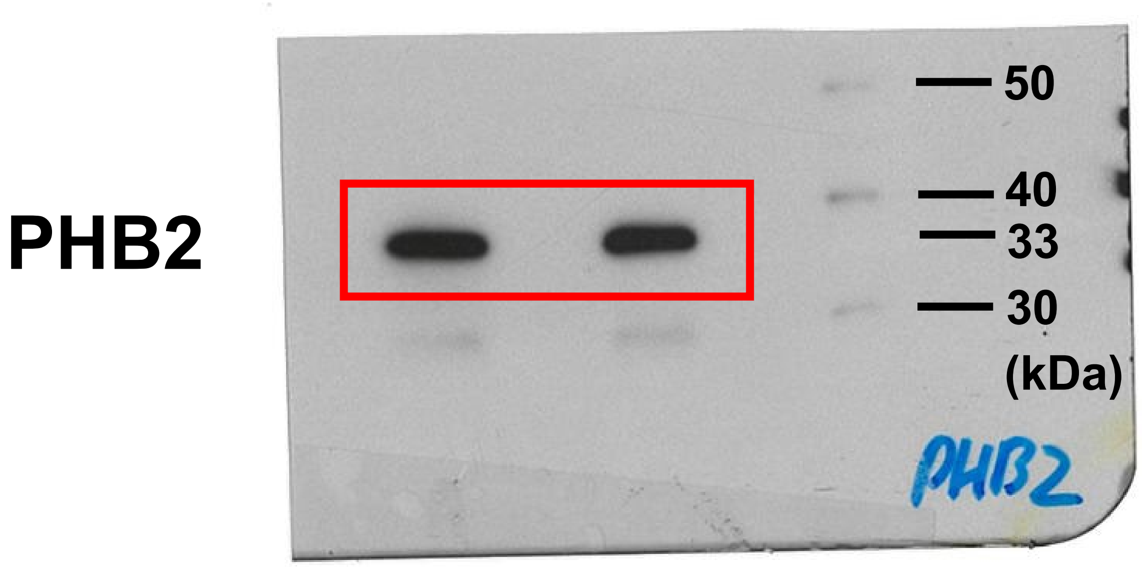

Uncropped western blots for Figure 2D

Molecular weights were estimated using MagicMark^{TM} XP Western Protein Standard (Thermo Fisher Scientific, Waltham, MA, USA) and DynaMarker® Protein MultiColor Stable II (BioDynamics Laboratory Inc., Tokyo, Japan).

Supplementary Fig. 5

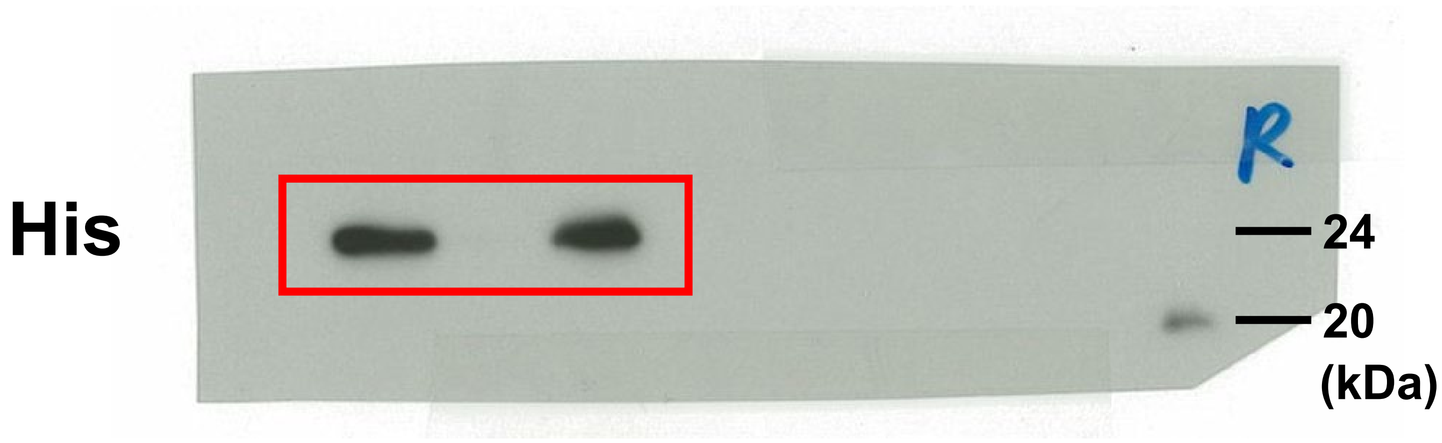

Uncropped western blots for Figure 2E

Molecular weights were estimated using MagicMark™ XP Western Protein Standard (Thermo Fisher Scientific, Waltham, MA, USA) and DynaMarker® Protein MultiColor Stable II (BioDynamics Laboratory Inc., Tokyo, Japan).

Supplementary Fig. 6

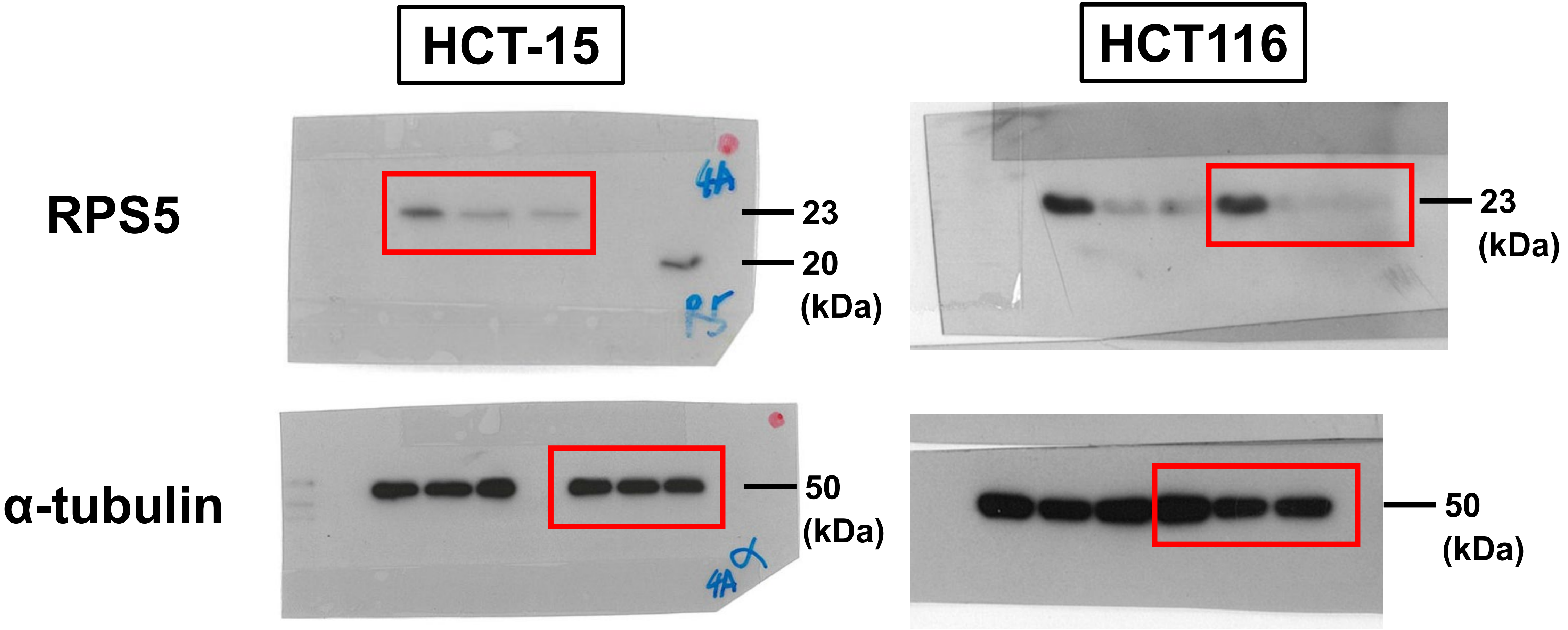

Uncropped western blots for Figure 4A

Molecular weights were estimated using MagicMark™ XP Western Protein Standard (Thermo Fisher Scientific, Waltham, MA, USA) and DynaMarker® Protein MultiColor Stable II (BioDynamics Laboratory Inc., Tokyo, Japan).

Supplementary Fig. 7

cyclin D1

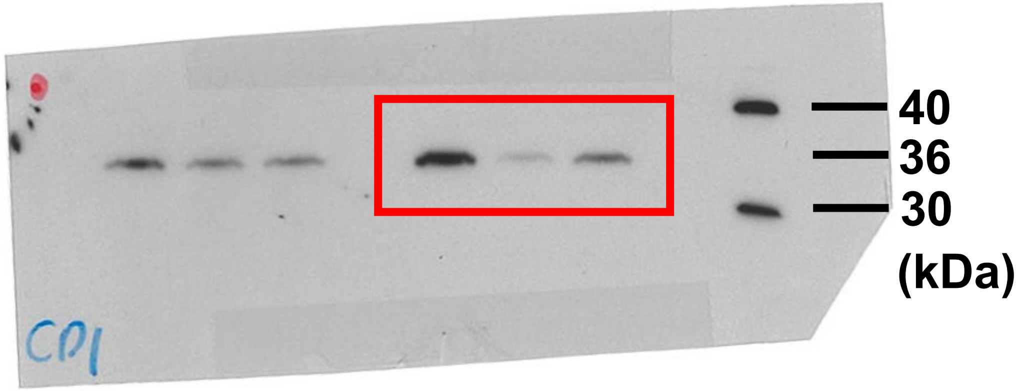

α-tubulin

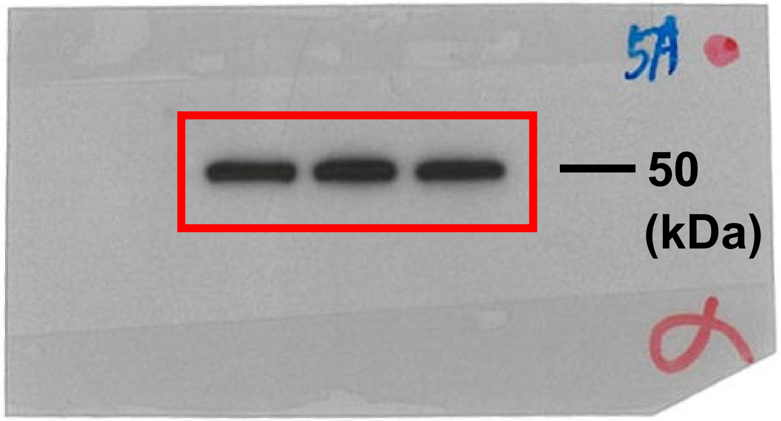

Uncropped western blots for Figure 5A

Molecular weights were estimated using MagicMark™ XP Western Protein Standard (Thermo Fisher Scientific, Waltham, MA, USA) and DynaMarker® Protein MultiColor Stable II (BioDynamics Laboratory Inc., Tokyo, Japan).

Supplementary Fig. 8

cyclin D1

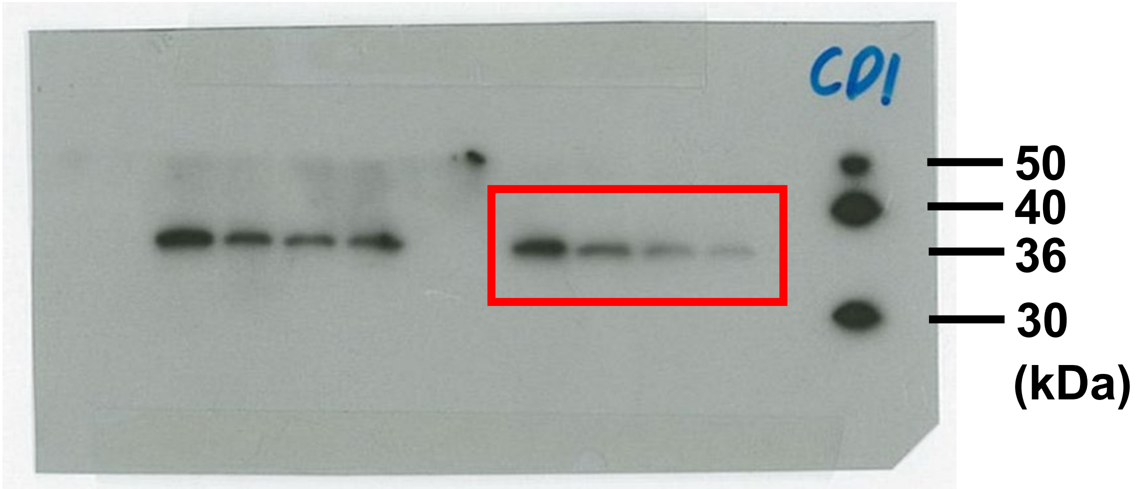

α-tubulin

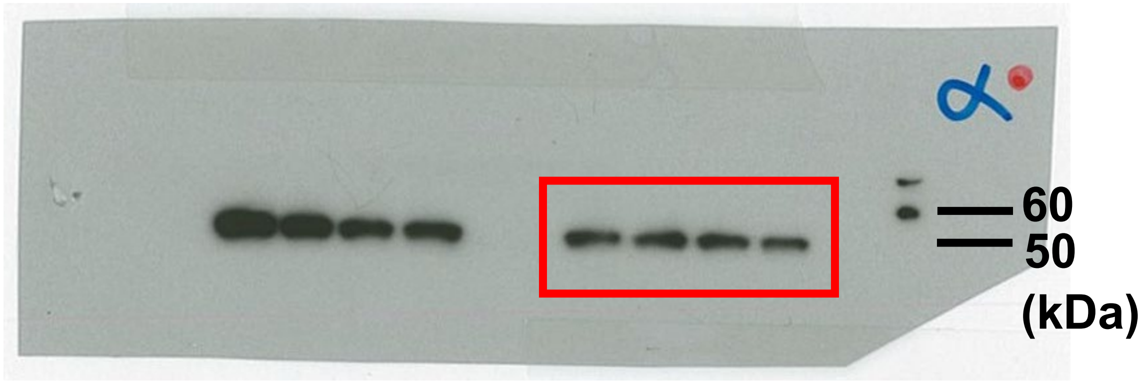

Uncropped western blots for Figure 5B

Molecular weights were estimated using MagicMark™ XP Western Protein Standard (Thermo Fisher Scientific, Waltham, MA, USA) and DynaMarker® Protein MultiColor Stable II (BioDynamics Laboratory Inc., Tokyo, Japan).

Supplementary Fig. 9

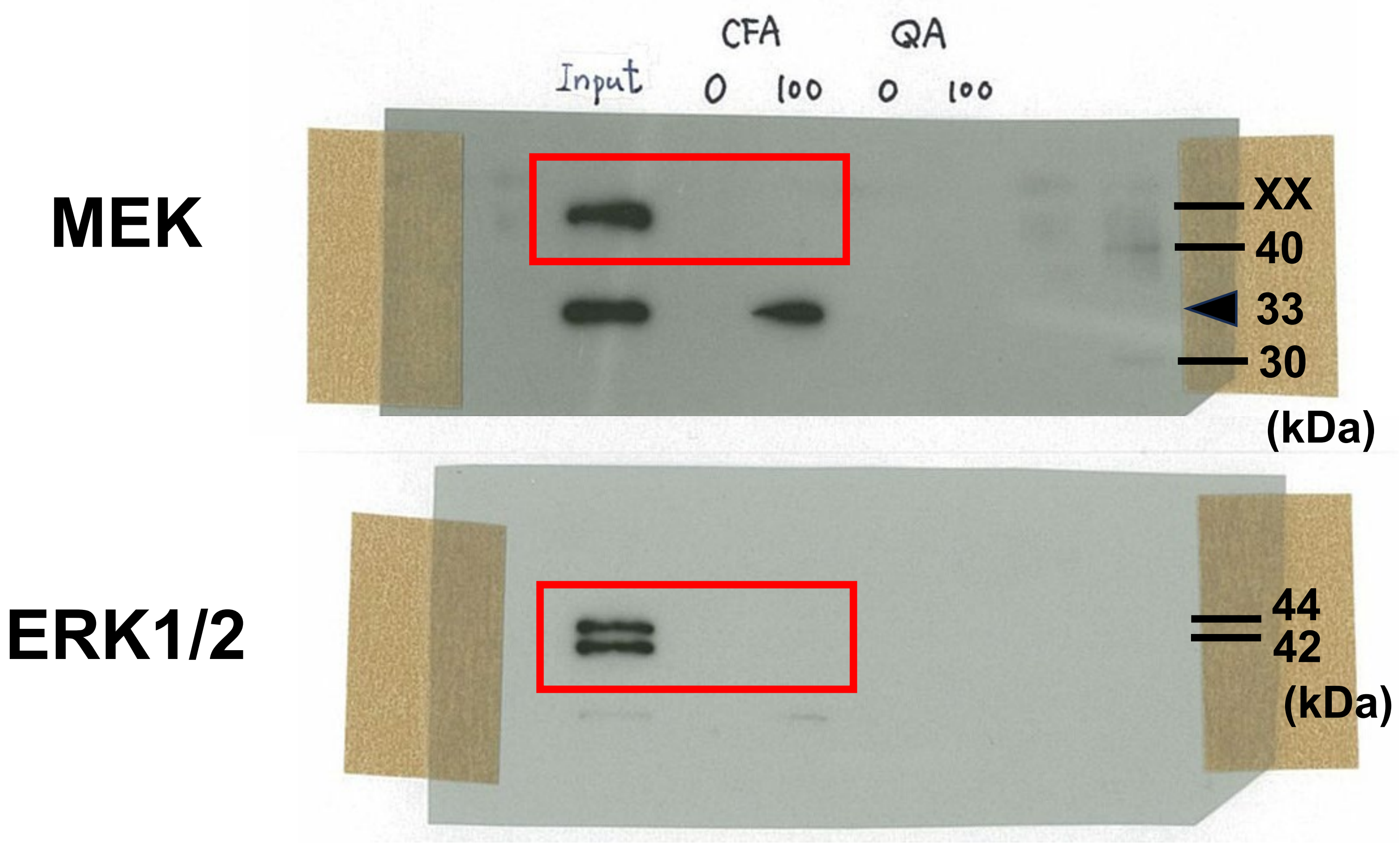

Uncropped western blots for Supplementary Fig. 1

Molecular weights were estimated using MagicMark^{TM} XP Western Protein Standard (Thermo Fisher Scientific, Waltham, MA, USA) and DynaMarker® Protein MultiColor Stable II (BioDynamics Laboratory Inc., Tokyo, Japan).
The arrow indicates the residual PHB2 signal after stripping and reprobing.

Supplementary Fig. 10

p-ERK1/2
(Thr202/Tyr204)

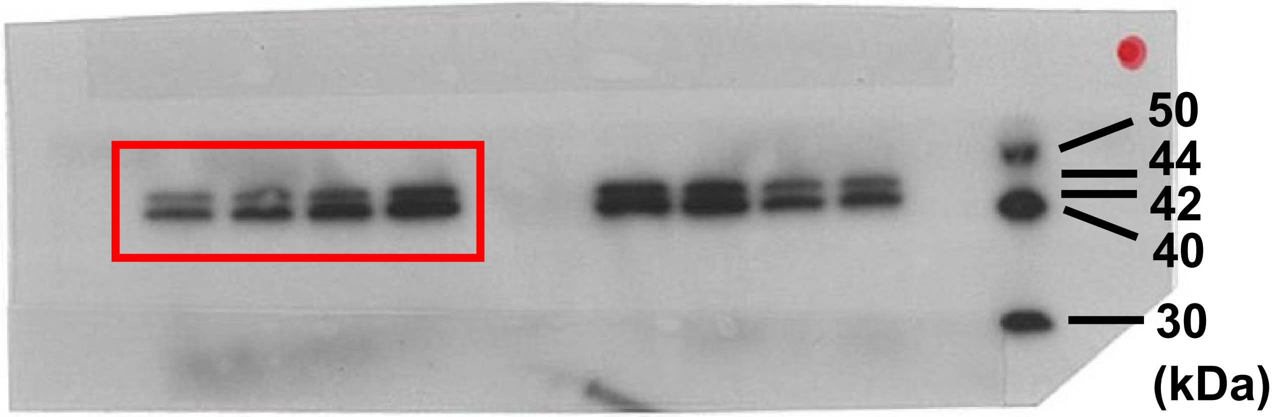

ERK1/2

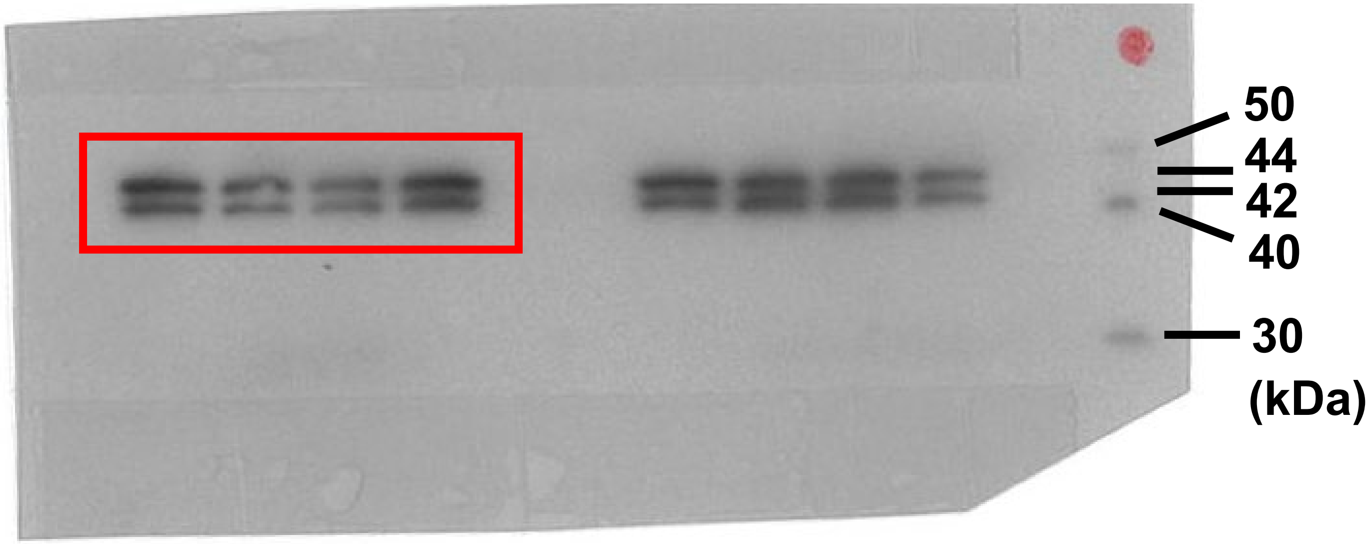

Uncropped western blots for Supplementary Fig. 2

Molecular weights were estimated using MagicMark™ XP Western Protein Standard (Thermo Fisher Scientific, Waltham, MA, USA) and DynaMarker® Protein MultiColor Stable II (BioDynamics Laboratory Inc., Tokyo, Japan).

Supplementary Fig. 11
